# Optimizing the Chemical Composition of Brake Shoes According to the Hardness Recommended by the Product Standard

**DOI:** 10.3390/ma16206797

**Published:** 2023-10-21

**Authors:** Erika Ardelean, Flavius Bucur, Corneliu Birtok-Băneasă, Ana Socalici, Marius Ardelean, Adina Budiul Berghian

**Affiliations:** Faculty Engineering of Hunedoara, Politehnica University Timisoara, Revolutiei No. 5, 331128 Hunedoara, Romania; corneliu.birtok@fih.upt.ro (C.B.-B.); virginia.socalici@fih.upt.ro (A.S.); marius.ardelean@fih.upt.ro (M.A.); adina.budiul@fih.upt.ro (A.B.B.)

**Keywords:** brake shoes, cast iron, hardness

## Abstract

Optimizing the chemical composition of phosphorus cast iron for the manufacture of brake shoes, which are used in the rolling stock, is based on specific tests, and the results must be within the specified limits of railway standards. This paper presents the processing of data taken from an economic agent producing brake shoes—with chemical composition and hardness values determined through the Brinell method—as well as optimizing these data by obtaining optimal variation intervals. These technological intervals are useful in the case of foundries in order to obtain superior products from a qualitative point of view.

## 1. Introduction

Brake shoes for rolling stock are made of P10-type phosphorus cast iron. [1,2,3]. They wear out relatively quickly, have a speed-dependent coefficient of friction, and sparks can occur during their operation in braking, leading to the strong heating of the shoe [2]. A quality assessment of the clogs in operation is made on the basis of the following criteria [1,4]: the stability of the braking road, minimum specific wear, maximum thermal load capacity, minimum sensitivity to breakage, and a low tendency to produce sparks.

Brake shoes (types S1, S2, and S3) are obtained by casting from a phosphorus cast iron and are intended for towed rolling stock. Phosphorus gray cast iron in its composition is perlite cast iron with an increased phosphorus content. A phosphorous ternary eutectic system, called steadite, consisting of perlite, cementite, and iron phosphide Fe_3_P [1,5], is formed in the cast iron structure. The maximum resistance of cast iron corresponds to the perlite metal mass, and perlite–cementite and phosphorus cast iron have the highest hardness. The highest vibration damping capacity belongs to ferritic metal mass at the graphite’s pick point [5].

During the solidification of phosphorus cast iron, phosphorus forms ternary phosphorus eutectic, which comprises a solid solution (Fe–C–P), iron phosphide (Fe_3_P), and cementite (Fe_3_C) [4,5]. Due to a low solidification temperature (below 1000 °C), phosphorus eutectic is distributed to the limits of grains in the form of isolated separations, constituting a discontinuous or continuous network depending on the phosphorus content [5,6,7,8]. The tendency to form a phosphorus eutectic is all the more accentuated when the silicon and carbon contents are higher, and the rate of cooling for the cast iron is lower. This is explained by the fact that silicon and carbon reduce the solubility and rate of phosphorus diffusion in the solid solution and, therefore, increase its tendency to segregate. The same effect can have a reduced cooling speed [1,6,7,8,9,10,11].

The maximum surface occupied by the phosphorus eutectic in cast iron is 15%, and the maximum dimension of its separations is 1 mm. The phosphorus eutectic is characterized by a high hardness (500–600 HB) and high fragility, which explains why it has a great influence on the properties of cast iron. The amount of phosphorus eutectic in cast iron is directly dependent on the phosphorus content, which is also influenced by factors that increase the tendency of phosphorus to segregate [1,12,13]. When the phosphorus content increases, there is an increase in the tensile strength of the cast iron due to graphite and perlite finishing. However, this increase in the proportion of phosphorus eutectic leads to the fragility of the cast iron so that at a certain value of phosphorus content, there is a decrease in resistance. The critical value depends mostly on the presence of elements that favor the segregation of phosphorus in the composition of cast iron. Thus, when the carbon content increases, there is a decrease in the phosphorus content, at which the fragility of phosphorus eutectic [1,5,6,13] is manifested.

The presence and increase in the proportion of phosphorous eutectic (a hard constituent) makes the hardness of the cast iron continuously increase. The fragility effect of the phosphorus eutectic is highlighted, in particular, by the fact that the resilience of cast iron decreases continuously with the increase in the phosphorus content [5].

With respect to cast iron, microporosity must be taken into account. This is generally specific to gray cast iron due to contraction microcavities that occur in intercellular spaces but is strongly accentuated in the case of a phosphorus cast iron, when these spaces solidify at low temperatures, resulting in the separations of the phosphorous eutectic [5,12,14].

Another feature of the eutectic solidification of phosphorus gray cast iron is the presence of phosphorus-rich areas inside eutectic cells in their central part. In such cast irons, the eutectic cells are found “as a spongy material, impregnated with phosphorus-rich liquid” [5,8,12,14].

Eutectic cells in a phosphorus gray cast iron have the following specific structures [5,8,12,14]:

- Arched graphite slides of different sizes in an orderly arrangement with very fine blades in the center, fine blades in the intermediate areas, and coarse slides outside the eutectic cells, respectively;
- Graphite slides in intercellular spaces, which also belong to neighboring cells;
- Separations of the phosphorous eutectic (common ternary) in intercellular spaces;
- Phosphorus-rich areas in the central part of the eutectic cells are associated with very fine graphite.

In general, there are several factors influencing the formation of contraction porosity, specific to the central areas of cast iron parts, including, namely, the nature of the materials used in the load of the elaboration unit, the chemical composition of the cast iron, the thermal elaboration regime, graphitizing modification, and rigidity of forms [15].

For cast iron brake shoes, it is known that at high temperatures during braking (800–900 °C), eutectic phosphide in the immediate vicinity of eutectic cells melts and flows around the austenitic crystals of the rough surface of the shoe. This increases the surface of the wheel contact shoe and develops greater friction [11,16,17,18,19]. The phosphorus content of cast iron is important, but the influence of other components on the metallographic structure must not be neglected.

The technical quality conditions and main dimensions of P10 phosphorus cast iron brake shoes for normal gauge railway rolling stock are specified in Task No. 1/SFMR/SDT/2000/Brake Shoes for the motor and towed rolling stock, as approved by the Romanian Railway Authority [2]. The manufacture of very important brake shoes relies on the influence of various chemical elements in the cast iron structure to prevent obtaining phosphorus cast iron that is too hard, which would prematurely wear the vehicle wheel bandage rail, or too soft, which would increase the consumption of brake shoes due to premature wear [20,21].

## 2. Experimental Research

Experiments were conducted to study the influence of the chemical composition of cast iron on the hardness of the brake shoes intended for the rolling stock.

Experimental research was performed in a foundry that had induction electric furnaces, an automatic air-pressing machine model HFP 2, and a semi-automatic casting line for the manufacture of rolling stock brake shoes. Research was performed on 70 phosphorus cast iron charges, from which 2 types of brake shoes (type S1 and S2) were poured [3,21].

The process to construct brake shoes consists of obtaining phosphorus cast iron and casting it into shapes, the technological flow of which is composed of the preparation of training mixtures shapes, the elaboration and casting of phosphorus cast iron, and debating and cleaning the brake shoes. The composition of the metal load on the experimental batches was 10–30%, first infused with cast iron, 20–60% cast iron waste (used shoes), and 20–30% steel waste. Dimensional load preparation has a special role in the elaboration process; it must be advanced and prepared. The resultant liquid cast iron was cast by gravity into temporary shapes (the casting temperature varied between 1470 and 1485 °C). A semi-automatic forming–casting line was used, and the casting form included a battery of 8 brake shoes. The cooling of the shoes was performed in the air at the temperature of the foundry. The brake shoes were approved, and the activities of supplying railway products were authorized by the Romanian Railway Authority, AFER. 

The quality control of the brake shoes was conducted according to specification no.1/SFMR/SDT/2000 [2]. Data on the standard chemical composition of the cast iron is given in Table 1. The average chemical composition of the experimental batches is shown in Table 2. When analyzing the experimental data, a classification of the chemical composition in the product standard was obtained. It also presented the values calculated for the Si/C ratio and C_equivalent_ (in relation to 1 [22]).

C_equivalent_ = %C_t_ + 0.31%Si − 0.027%Mn + 0.33%P + 0.4%S (1)

**Figure 1:**
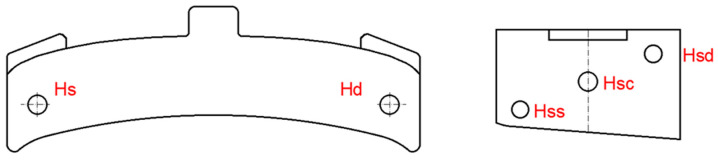
The areas for determining the hardness of the brake shoes.

The Brinell method (specified in SR EN ISO 6506-1:2015 [23]) was used to determine the hardness of experienced brake shoes, and the measurements were performed in the areas shown in Figure 1 with the following notations:

- Hs and Hd—at one point at each end of the shoe, on its face, after removing 2 mm of material, by grinding or grinding;
- Hss, Hsc, and Hsd—at three points located diagonally on the surface of a sample and obtained by cross-sectioning the broken clogs during the shock test.

Experimental data on the variation in the hardness of the brake shoes determined both on the surface of the shoe (HBs, HBd) and, in its section (HBss, HBsc, HBsd), the hardness measurement points specified in the standard are shown in Table 3.

The data analysis showed that there were small variations in the hardness measured on the side surface and in the cross-section of the brake shoe. This was due to the narrow variation in the chemical composition of the brake shoes. The values of hardness obtained during industrial scale experiments fell within the range of 197–255 HB, which is in accordance with international standards UIC 830-O [24].

The data obtained in industrial practice were processed in the Matlab calculation program (version R2016a). Hardness variation charts were made for carbon, manganese, phosphorus, and silicon for all points where the hardness measurement was performed. Using Matlab software, three-dimensional graphs with regression surfaces and level curves were built for the variation in hardness depending on the chemical composition concerned, and equations of ordinary variation II were generated as follows:

(2) $$z=a\left(1\right)\cdot x^{2}+a\left(2\right)\cdot y^{2}+a\left(3\right)\cdot x\cdot y+a\left(4\right)\cdot x+a\left(5\right)\cdot y+a\left(6\right)$$

In Figure 2, Figure 3, Figure 4, Figure 5, Figure 6, Figure 7, Figure 8, Figure 9, Figure 10, Figure 11, Figure 12, Figure 13, Figure 14, Figure 15, Figure 16, Figure 17, Figure 18, Figure 19, Figure 20, Figure 21, Figure 22, Figure 23, Figure 24, Figure 25, Figure 26, Figure 27, Figure 28, Figure 29, Figure 30 and Figure 31, the regression surfaces and level curves for the variation in the average hardness in the section are presented according to the elements of the chemical composition of cast iron (C, Mn, Si, S, P). These results are also presented via correlation equations.

A metallographic structural examination was performed according to the specifications [2] using an inverted metallographic microscope type GX41 (by OLYMPUS Corporation, Tokyo, Japan) alongside the following:

- A microscope with a thickness of G of 100× on polished and not chemically attacked specimens for graphite visualization;
- A microscope with G-trushes in the order of 200×, 50 or 100×, 25 or 50× on the same specimens attacked with Nital alcoholic acid solution (4%) to examine the structure with a normal attack time, checking perlite and ferrite and with a prolonged attack duration, to visualize the phosphorus eutectic network.

**Figure 2:**
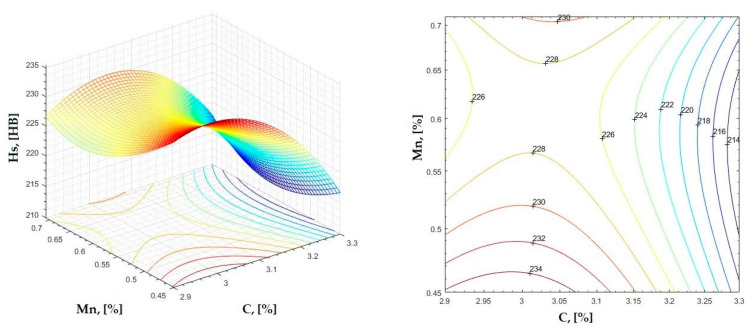
Regression area and level curves for Hs = f(C, Mn).

$$\mathrm{Hs}=-187.40\cdot C^{2}+300.30\cdot {\mathrm{Mn}}^{2}+85.60\cdot C\cdot \mathrm{Mn}+1079.40\cdot C-620.0\cdot \mathrm{Mn}-1210.80$$

**Figure 3:**
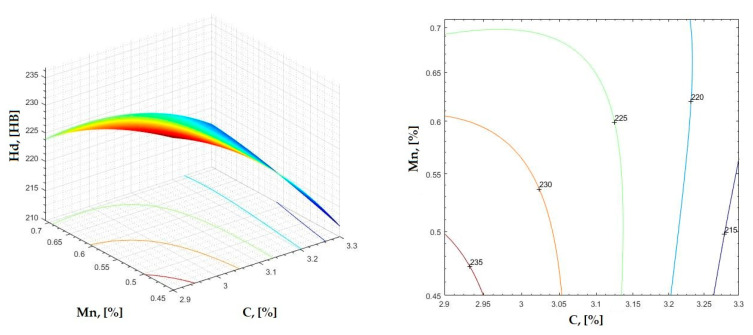
Regression area and level curves for Hd = f(C, Mn).

$$\mathrm{Hd}=-73.23\cdot C^{2}-54.24\cdot {\mathrm{Mn}}^{2}+176.45\cdot C\cdot \mathrm{Mn}+311.85\cdot C-498.69\cdot \mathrm{Mn}-46.50$$

**Figure 4:**
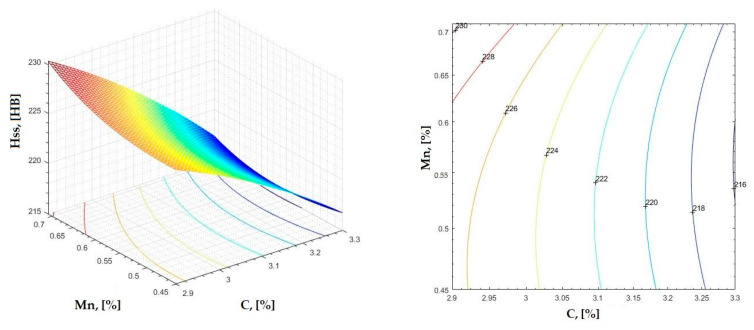
Regression area and level curves for Hss = f(C, Mn).

$$\mathrm{Hss}=-13.78\cdot C^{2}+65.66\cdot {\mathrm{Mn}}^{2}-32.17\cdot C\cdot \mathrm{Mn}+75.68\cdot C+32.42\cdot \mathrm{Mn}+136.91$$

**Figure 5:**
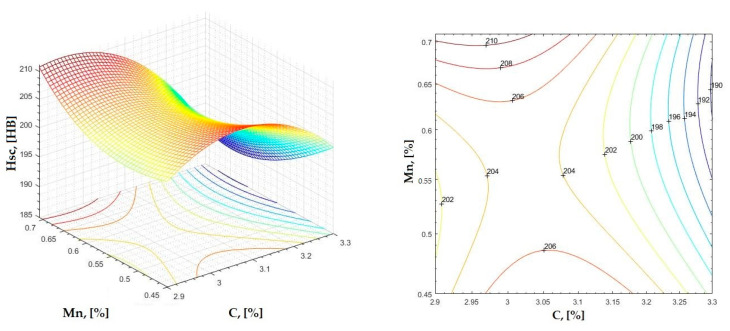
Regression area and level curves for Hsc = f(C, Mn).

$$\mathrm{Hsc}=-166.9\cdot C^{2}+253.5\cdot {\mathrm{Mn}}^{2}-159.9\cdot C\cdot \mathrm{Mn}+1098.3\cdot C+200.6\cdot \mathrm{Mn}-1511.4$$

**Figure 6:**
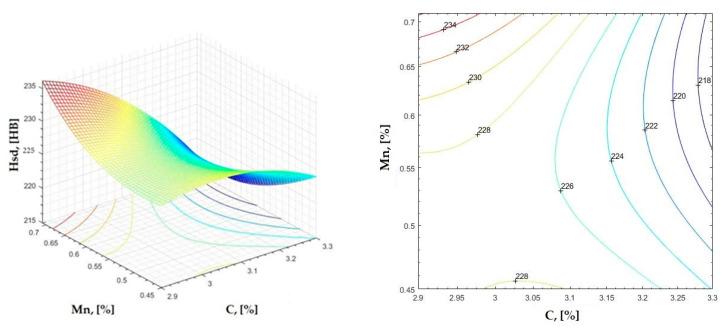
Regression area and level curves for Hsd = f(C, Mn).

$$\mathrm{Hsd}=-62.30\cdot C^{2}+180.90\cdot {\mathrm{Mn}}^{2}-151.53\cdot C\cdot \mathrm{Mn}+446.81\cdot C+264.94\cdot \mathrm{Mn}-502.87$$

**Figure 7:**
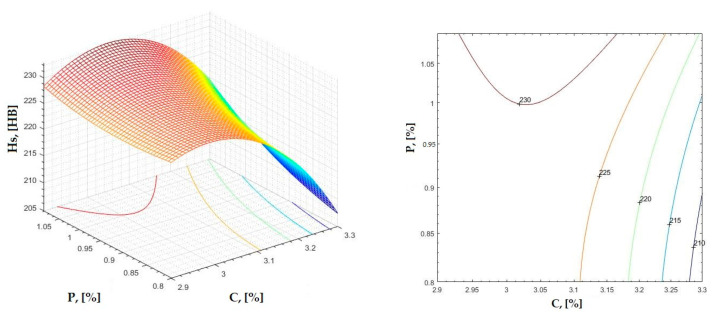
Regression area and level curves for Hs = f(C, P).

$$\mathrm{Hs}=-211.70\cdot C^{2}+68.50\cdot P^{2}+90.70\cdot C\cdot P+1191.40\cdot C-386.90\cdot P-1393.30$$

**Figure 8:**
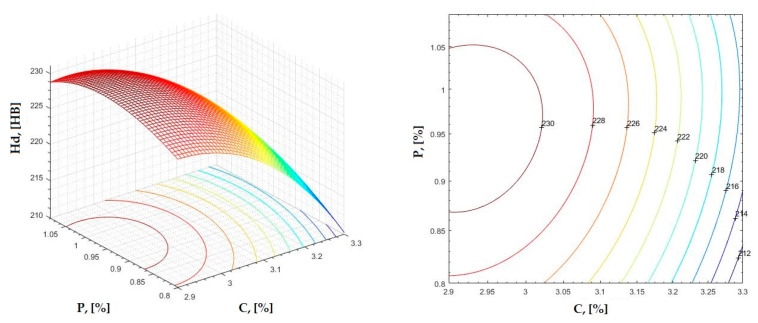
Regression area and level curves for Hd = f(C, P).

$$\mathrm{Hd}=-108.30\cdot C^{2}-135.59\cdot P^{2}+28.33\cdot C\cdot P+605.08\cdot C+177.67\cdot P-737.30$$

**Figure 9:**
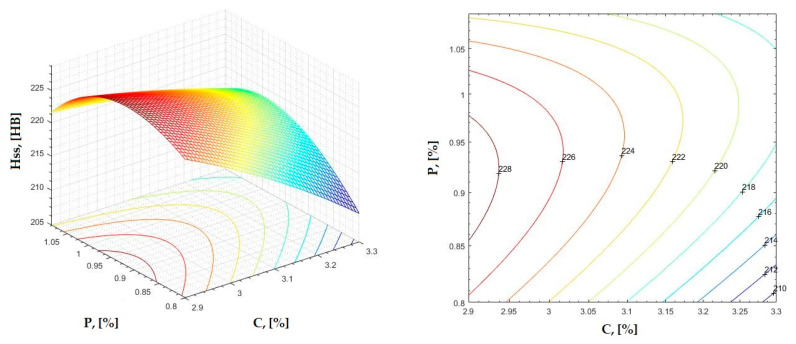
Regression area and level curves for Hss = f(C, P).

$$\mathrm{Hss}=-13.71\cdot C^{2}-241.60\cdot P^{2}+99.89\cdot C\cdot P-36.37\cdot C+152.90\cdot P+247.01$$

**Figure 10:**
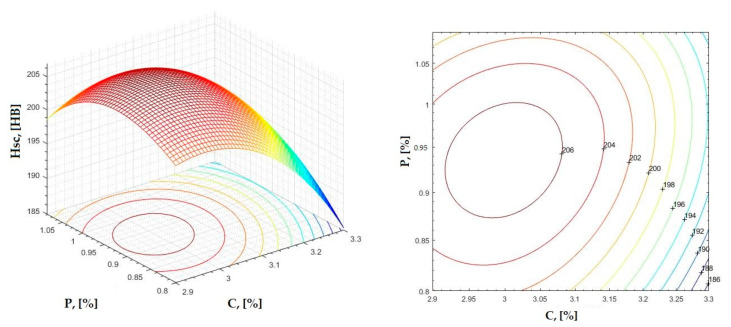
Regression area and level curves for Hsc = f(C, P).

$$\mathrm{Hsc}=-151.90\cdot C^{2}-246.60\cdot P^{2}+79.90\cdot C\cdot P+836.3\cdot C+222.8\cdot P-1151.60$$

**Figure 11:**
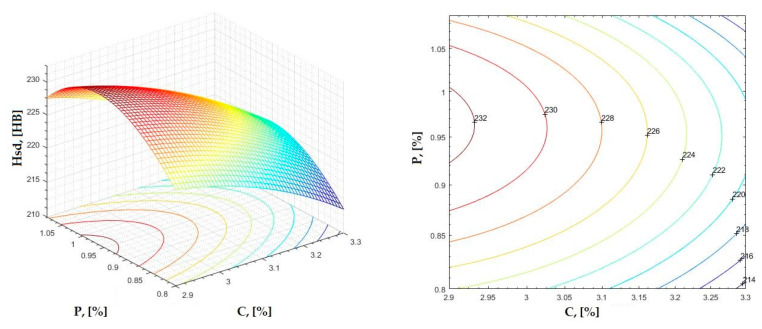
Regression area and level curves for Hsd = f(C, P).

$$\mathrm{Hsd}=-36.91\cdot C^{2}-314.77\cdot P^{2}-21.08\cdot C\cdot P+218.99\cdot C+667.86\cdot P-384.41$$

**Figure 12:**
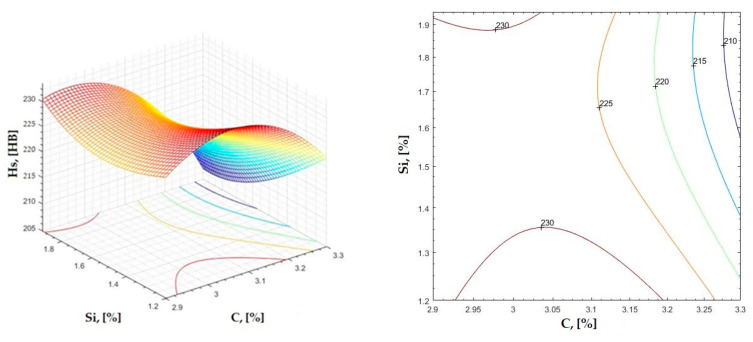
Regression area and level curves for Hs = f(C, Si).

$$\mathrm{Hs}=-209.30\cdot C^{2}+31.50\cdot {\mathrm{Si}}^{2}-57.50\cdot C\cdot \mathrm{Si}+1350.20\cdot C+70.7\cdot \mathrm{Si}-1857.20$$

**Figure 13:**
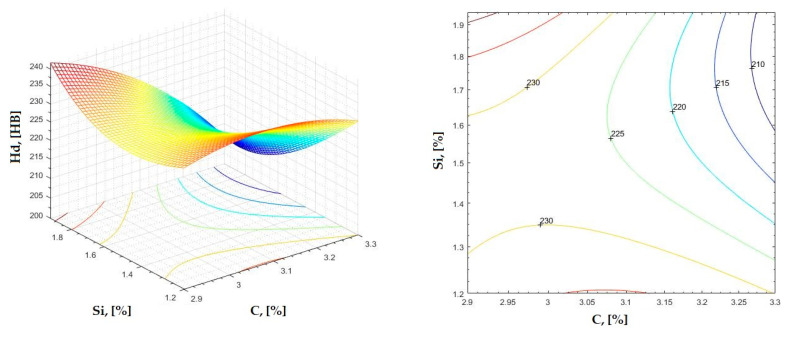
Regression area and level curves for Hd = f(C, Si).

$$\mathrm{Hd}=-108.10\cdot C^{2}+57.40\cdot {\mathrm{Si}}^{2}-111.50\cdot C\cdot \mathrm{Si}+798.60\cdot C+156.3\cdot \mathrm{Si}-1057.00$$

**Figure 14:**
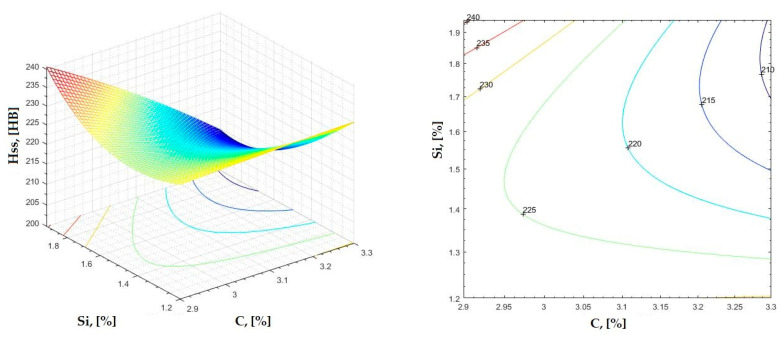
Regression area and level curves for Hss = f(C, Si).

$$\mathrm{Hss}=-5.10\cdot C^{2}+53.58\cdot {\mathrm{Si}}^{2}-110.99\cdot C\cdot \mathrm{Si}+169.21\cdot C+170.68\cdot \mathrm{Si}-114.69$$

**Figure 15:**
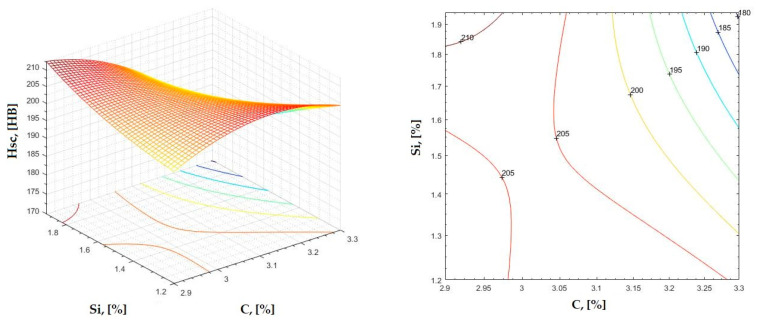
Regression area and level curves for Hsc = f(C, Si).

$$\mathrm{Hsc}=-144.20\cdot C^{2}+11.80\cdot {\mathrm{Si}}^{2}-125.30\cdot C\cdot \mathrm{Si}+1053.70\cdot C+343.00\cdot \mathrm{Si}-1635.00$$

**Figure 16:**
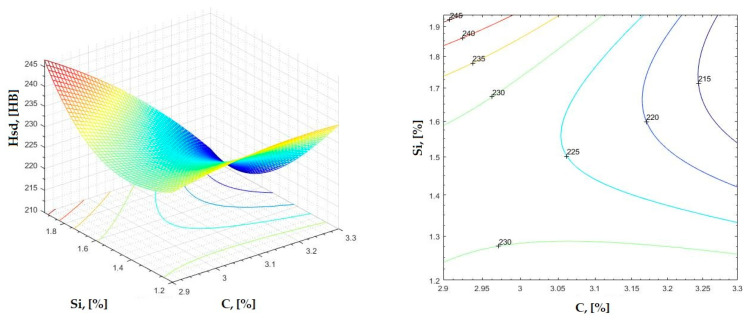
Regression area and level curves for Hsd = f(C, Si).

$$\mathrm{Hsd}=-37.43\cdot C^{2}+68.15\cdot {\mathrm{Si}}^{2}-128.68\cdot C\cdot \mathrm{Si}+394.98\cdot C+180.50\cdot \mathrm{Si}-466.55$$

**Figure 17:**
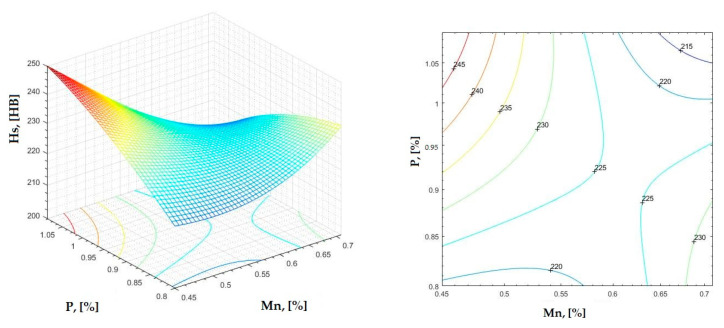
Regression area and level curves for Hs = f(Mn, P).

$$\mathrm{Hs}=379.28\cdot {\mathrm{Mn}}^{2}-160.39\cdot P^{2}-738.31\cdot \mathrm{Mn}\cdot P+210.26\cdot \mathrm{Mn}+742.36\cdot P-177.73$$

**Figure 18:**
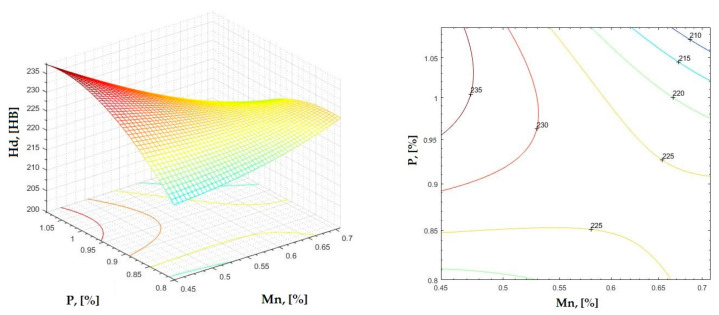
Regression area and level curves for Hd = f(Mn, P).

$$\mathrm{Hd}=75.86\cdot {\mathrm{Mn}}^{2}-305.46\cdot P^{2}-550.91\cdot \mathrm{Mn}\cdot P+387.27\cdot \mathrm{Mn}+891.51\cdot P-290.67$$

**Figure 19:**
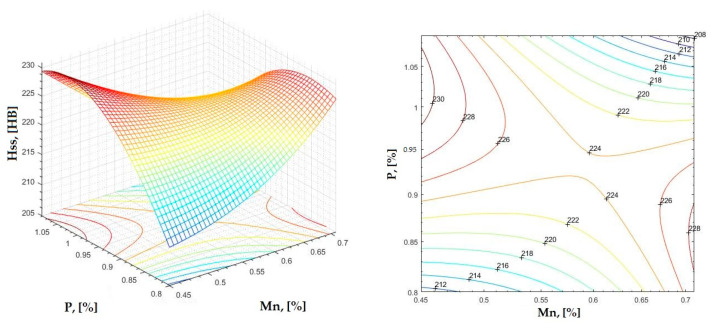
Regression area and level curves for Hss = f(Mn, P).

$$\mathrm{Hss}=160.20\cdot {\mathrm{Mn}}^{2}-383.70\cdot P^{2}-506.80\cdot \mathrm{Mn}\cdot P+281.60\cdot \mathrm{Mn}+1015.90\cdot P-332.60$$

**Figure 20:**
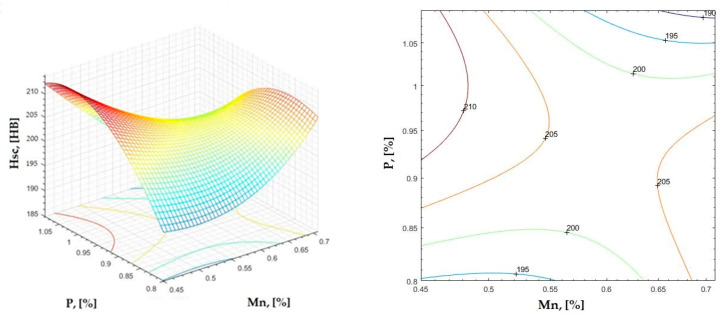
Regression area and level curves for Hsc = f(Mn, P).

$$\mathrm{Hsc}=313.80\cdot {\mathrm{Mn}}^{2}-402.80\cdot P^{2}-498.00\cdot \mathrm{Mn}\cdot P+86.40\cdot \mathrm{Mn}+1046.20\cdot P-307.70$$

**Figure 21:**
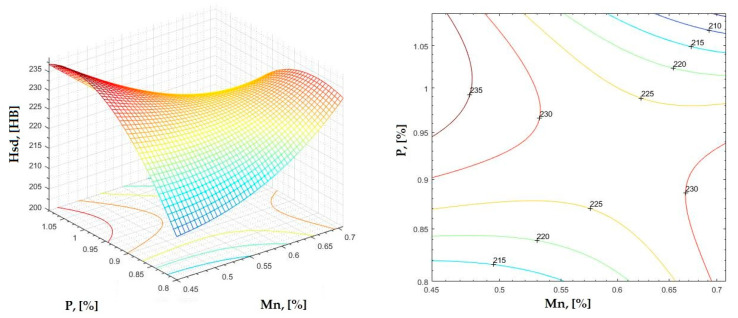
Regression area and level curves for Hsd = f(Mn, P).

$$\mathrm{Hsd}=251.60\cdot {\mathrm{Mn}}^{2}-536.50\cdot P^{2}-728.80\cdot \mathrm{Mn}\cdot P+375.30\cdot \mathrm{Mn}+1433.70\cdot P-550.70$$

**Figure 22:**
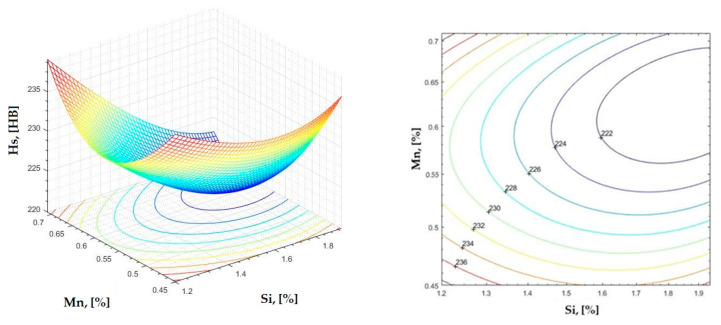
Regression area and level curves for Hs = f(Si, Mn).

$$\mathrm{Hs}=26.10\cdot {\mathrm{Si}}^{2}+492.39\cdot {\mathrm{Mn}}^{2}-72.40\cdot \mathrm{Si}\cdot \mathrm{Mn}-52.10\cdot \mathrm{Si}-481.72\cdot \mathrm{Mn}+419.62$$

**Figure 23:**
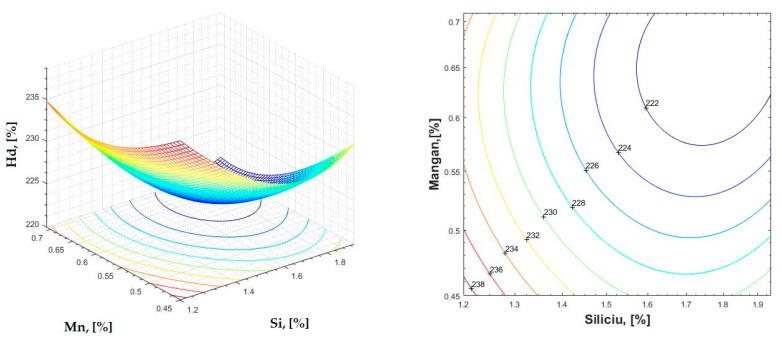
Regression area and level curves for Hd = f(Si, Mn).

$$\mathrm{Hd}=39.79\cdot {\mathrm{Si}}^{2}+197.86\cdot {\mathrm{Mn}}^{2}-31.29\cdot \mathrm{Si}\cdot \mathrm{Mn}-120.62\cdot \mathrm{Si}-207.63\cdot \mathrm{Mn}+396.63$$

**Figure 24:**
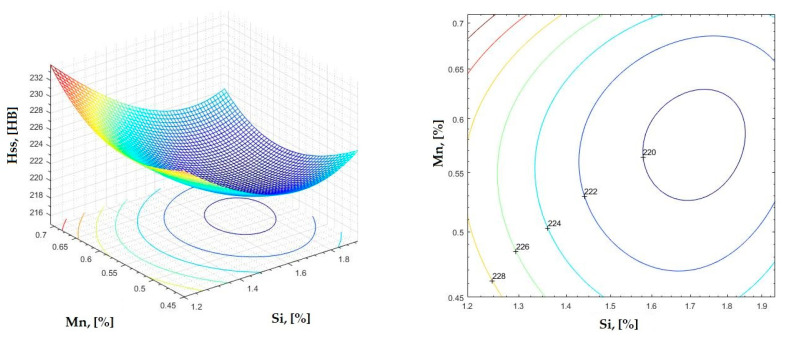
Regression area and level curves for Hss = f(Si, Mn).

$$\mathrm{Hss}=32.38\cdot {\mathrm{Si}}^{2}+228.05\cdot {\mathrm{Mn}}^{2}-28.16\cdot \mathrm{Si}\cdot \mathrm{Mn}-94.81\cdot \mathrm{Si}-215.08\cdot \mathrm{Mn}+362.80$$

**Figure 25:**
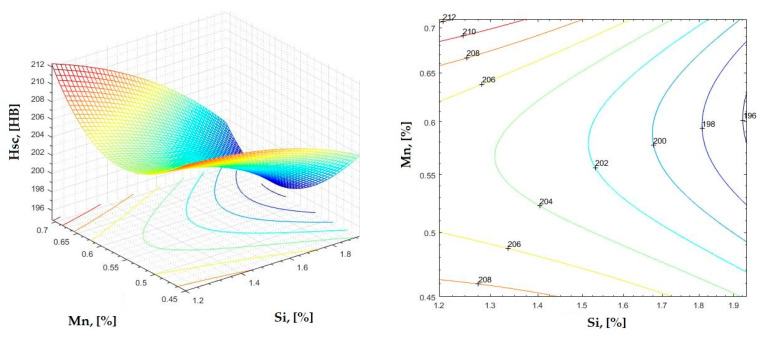
Regression area and level curves for Hsc = f(Si, Mn).

$$\mathrm{Hsc}=-6.59\cdot {\mathrm{Si}}^{2}+337.77\cdot {\mathrm{Mn}}^{2}-39.98\cdot \mathrm{Si}\cdot \mathrm{Mn}+31.80\cdot \mathrm{Si}-330.51\cdot \mathrm{Mn}+282.15$$

**Figure 26:**
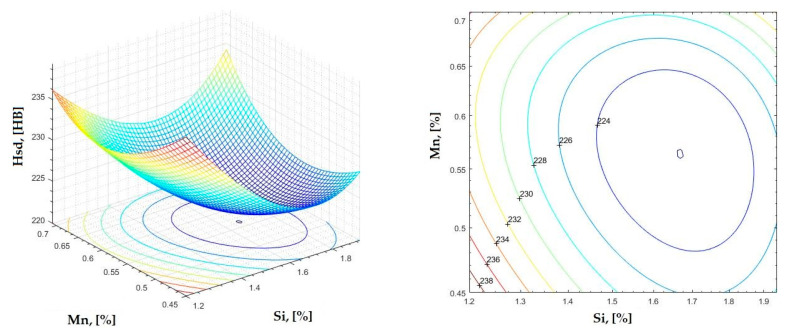
Regression area and level curves for Hsd = f(Si, Mn).

$$\mathrm{Hsd}=50.11\cdot {\mathrm{Si}}^{2}+306.88\cdot {\mathrm{Mn}}^{2}+48.51\cdot \mathrm{Si}\cdot \mathrm{Mn}-194.46\cdot \mathrm{Si}-426.88\cdot \mathrm{Mn}+504.42$$

**Figure 27:**
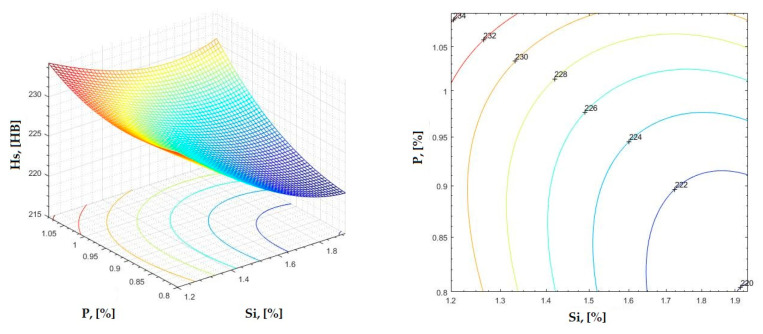
Regression area and level curves for Hs = f(Si, P).

$$\mathrm{Hs}=20.37\cdot {\mathrm{Si}}^{2}+100.50\cdot P^{2}+37.88\cdot \mathrm{Si}\cdot P-110.50\cdot \mathrm{Si}-226.80\cdot P+415.93$$

**Figure 28:**
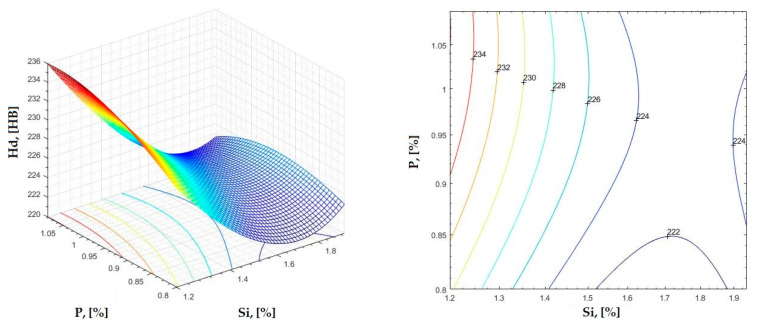
Regression area and level curves for Hd = f(Si, P).

$$\mathrm{Hd}=37.90\cdot {\mathrm{Si}}^{2}-82.64\cdot P^{2}-28.42\cdot \mathrm{Si}\cdot P-106.13\cdot \mathrm{Si}+210.24\cdot P+214.96$$

**Figure 29:**
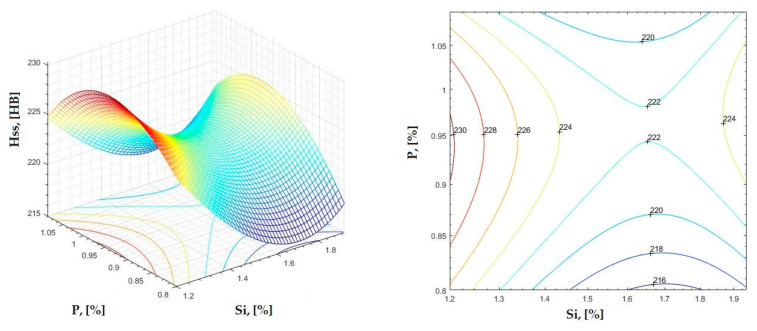
Regression area and level curves for Hss = f(Si, P).

$$\mathrm{Hss}=39.96\cdot {\mathrm{Si}}^{2}-244.02\cdot P^{2}+26.34\cdot \mathrm{Si}\cdot P-157.22\cdot \mathrm{Si}+426.10\cdot P+146.80$$

**Figure 30:**
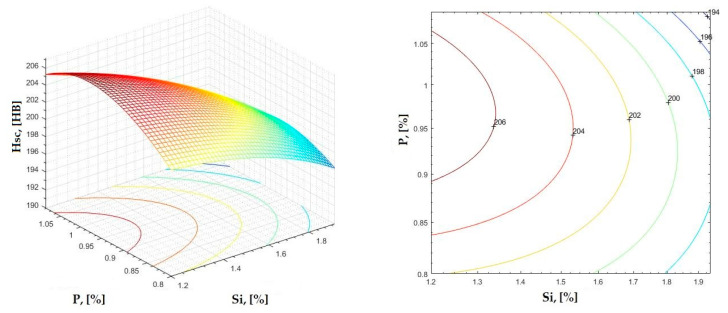
Regression area and level curves for Hsc = f(Si, P).

$$\mathrm{Hsc}=-6.92\cdot {\mathrm{Si}}^{2}-159.32\cdot P^{2}-27.54\cdot \mathrm{Si}\cdot P+35.82\cdot \mathrm{Si}+345.03\cdot P+21.45$$

**Figure 31:**
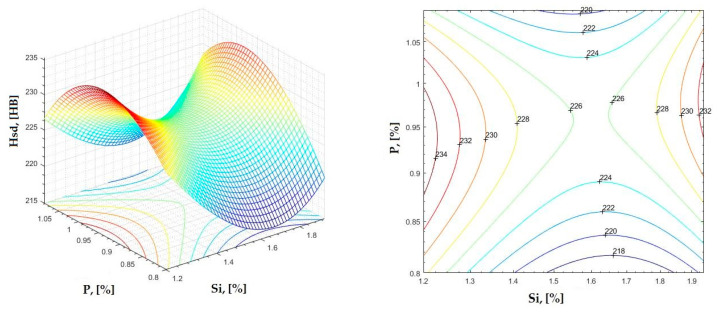
Regression area and level curves for Hsd = f(Si, P).

$$\mathrm{Hsd}=57.89\cdot {\mathrm{Si}}^{2}-370.57\cdot P^{2}+45.56\cdot \mathrm{Si}\cdot P-228.94\cdot \mathrm{Si}+639.05\cdot P+102.02$$

## 3. Results and Discussion

From the equations of the double correlation analysis, as expressed in the analytical and graphic form, a series of conclusions were obtained:

- The variation in independent parameters within the technological limits determines a variation in the dependent parameter with its location on or near a regression surface and in view of dispersion, deviation, and error.
- The graphic representations of grade II polynomial functions are surfaces with a stationary point that is either extreme (of the maximum or minimum) or saddle. Their coordinates are within the technological limits of variation for independent parameters, respectively, and within the limits provided by the specifications for the dependent parameter.
- The intersection of correlation surfaces with level planes (the parallels with the horizontal plane) and level curves were obtained, thus allowing the limits of variation in independent parameters to be established, obtaining a certain value for the dependent parameter, and finding that there are higher variation limits the independent parameters near the stationary point in cases where a saddle point exists. For each graphical representation, the subdomains in which the values for the dependent parameter are found can be indicated, which determines the variation limits for independent parameters.

By individually analyzing each surface—with the specific influence of two elements on the chemical composition of hardness—the optimal variation intervals of these elements were identified. These were as follows:

- The dependencies presented in Figure 2, Figure 3, Figure 4, Figure 5 and Figure 6 were determined as the following optimal variation intervals: 2.9–3.2%C and 0.63–0.68%Mn;
- The dependencies presented in Figure 7, Figure 8, Figure 9, Figure 10 and Figure 11 were determined as the following optimal variation intervals: 2.92–3.24%C and 0.84–1.05%P;
- The dependencies presented in Figure 12, Figure 13, Figure 14, Figure 15 and Figure 16 were determined as the following optimal variation intervals: 2.97–3.15%C and 1.3–1.82%Si;
- The dependencies presented in Figure 17, Figure 18, Figure 19, Figure 20 and Figure 21 were determined as the following optimal variation intervals: 0.47–0.65%Mn and 0.82–1.02%P;
- The dependencies presented in Figure 22, Figure 23, Figure 24, Figure 25 and Figure 26 were determined as the following optimal variation intervals: 1.24–1.82%Si and 0.48–0.68%Mn;
- The dependencies presented in Figure 27, Figure 28, Figure 29, Figure 30 and Figure 31 were determined as optimal variation intervals: 1.22–1.8%Si and 0.80–1.02%P.

Taking into account the analysis performed on each diagram, the optimal variation intervals for the analyzed elements resulted in the following: 2.97–3.15%C, 0.53–0.65%Mn, 1.3–1.8%Si, and 0.85–1.02%P. In terms of S content, it varied between 0.08 and 0.09%, and the residual elements did not exceed 0.2%.

From each batch, samples were taken and subjected to spectral analysis to determine their chemical composition. The metallographic examination of shoe cast iron was performed according to SR EN ISO 945-1:2019 [25], and the UIC 832 sheet and consisted of the analysis of four samples:

- A sample (without a chemical attack and with a thickness G of 100×) to identify the form of graphite separations, their character and the length of the lamellar graphite separations;
- A sample (Nital attack and a G-grosisment of at least 200×) to determine the perlite configuration and two samples (with Nital attack and a G-grosisment of 25× and 50×) for the base metal mass and phosphorus eutectic.

**Figure 32:**
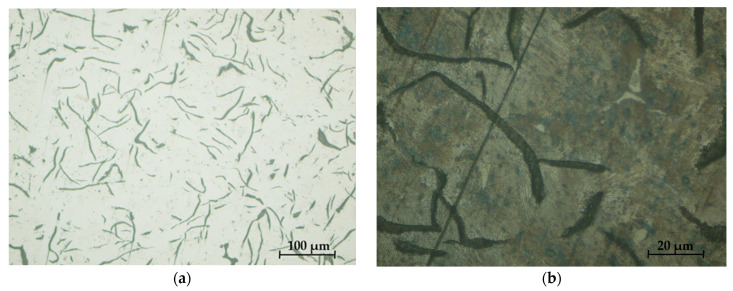

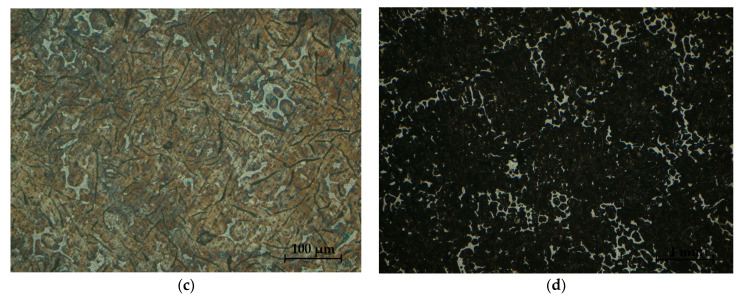
Sample microstructures—S1: (**a**) Graphite, unattacked 100×; (**b**) Lamellar pearlite, Nital attack 500×; (**c**) Base metal mass, Nital attack 100×; (**d**) Phosphorous eutectic, Nital attack 50×.

**Figure 33:**
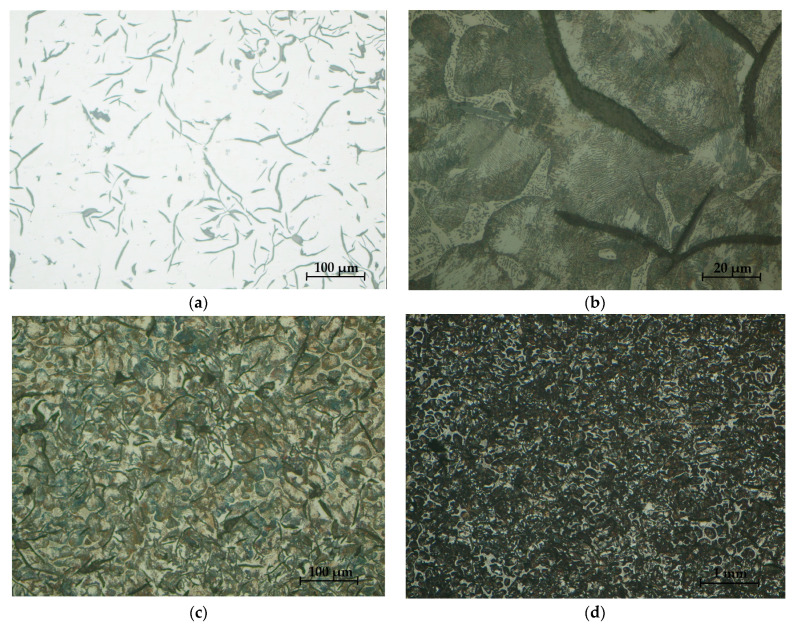
Sample microstructures—S2: (**a**) Graphite, unattacked 100×; (**b**) Lamellar pearlite, Nital attack 500×; (**c**) Base metal mass, Nital attack 100×; (**d**) Phosphorous eutectic, Nital attack 50×.

Figure 32 and Figure 33 present the microstructures of the analyzed samples.

The high content of phosphorus improves the friction behavior and wear of this cast iron; the star is formed in the structure. The structure consisting of perlite, graphite, and eutectic phosphorus (steadite) determined the resistance to wear and high fluidity. The properties of the cast iron were determined both by the nature of the metal mass and by the quantity, shape, and dimensions of graphite separations. The properties of resistance and plasticity increased with a reduction in the amount and size of graphite separations and their compaction.

The analysis of the microstructures presented in Figure 32 and Figure 33, respectively, produced the following results:

- The presence of lamellar graphite separations (at a proportion of 12.5% and 10%, respectively);
- The proportion of ferrite in the form of isolated islands was 1% and 5% of the area of the samples presented;
- A network of uniformly distributed phosphorus eutectic of approximately 15.5% and 12.5% was identified, respectively;
- A lamellar perlite configuration, with values of 71% and 72.5%, respectively, was obtained for the presented samples.

## 4. Industrial Verification of Results

In order to verify the results obtained, research was performed on the same technological flow for two charges, pouring S1-type brake shoes from quality cast iron-type P10-phosphorus cast iron.

The chemical composition of cast iron is presented in Table 4, and the values for hardness, determined under the same conditions, are presented in Table 5.

**Figure 34:**
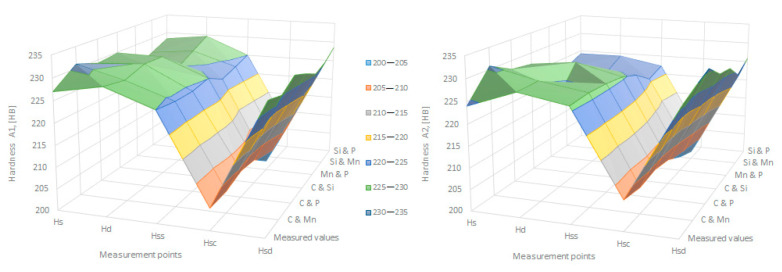
Comparison between the measured and calculated values for the hardening of charge A1 and A2.

To verify the applicability of the relationships obtained in Matlab and presented in point two, the values of hardness were calculated and compared with those determined in practice and presented in Table 5. These small resultant differences did not change the range in variation for hardness at the points required by these specifications, making it possible to use these relationships in industrial practice, as shown in Figure 34.

**Figure 35:**
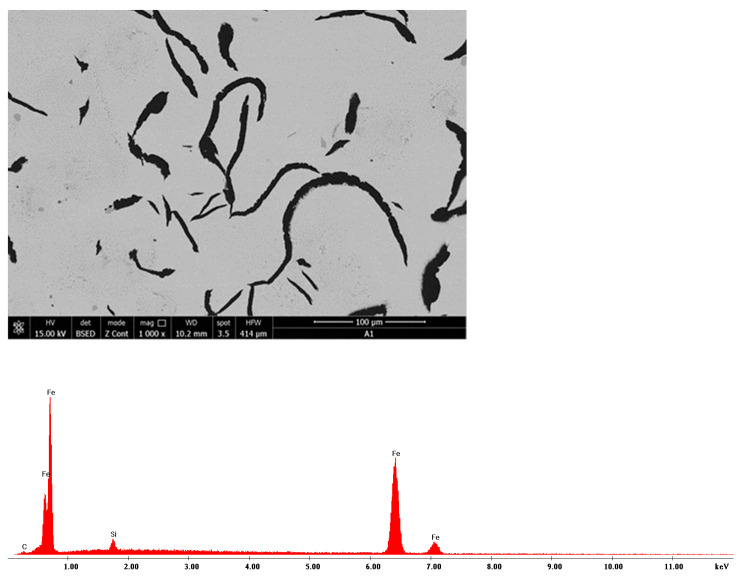
Microzone charge A1: EDS analysis and EDS spectrum, 1000×.

**Figure 36:**
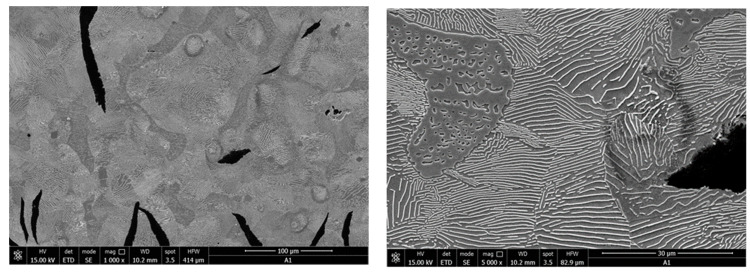
Sample microstructure area, Nital attack, 1000× and 5000×, respectively.

The Quanta FEG-250 SEM (by ELECMI, Zaragoza, Spain) was used for the micro-structural analysis of samples both with and without a reagent attack. The following microstructures relate to charge A1 and are presented as follows:

- In Figure 35, a sample of microzone (EDS) analysis and the related EDS spectrum is presented;
- In Figure 36, an area of the sample’s microstructure, Nital attack, with increases of 1000× and 5000×, respectively, is shown.

The microstructural analysis of samples attacked with 5% Nital indicates that the area occupied by perlite was between 45 and 65% (standard structure P50), and the analysis of the degree of perlite dispersion indicated fine slides (standard structure Pi 0.5). The phosphorus eutectic network was evenly distributed for the analyzed samples, and the proportion of ferrite in the form of isolated islands was less than 5% of the sample area.

## 5. Conclusions

From the analysis of experimental data taken from industrial practice and processed, the following conclusions were obtained:

- For the Matlab correlations, we considered, as a dependent parameter, the hardness (determined in two points on the surface of the brake shoe and in three points in the section of the respective brake shoe its average both on the surface and on its section) and the chemical composition of phosphorus cast iron from which the brake shoes were poured, was considered an independent parameter, thus obtaining double correlation equations;
- For all the correlations obtained, the correlation coefficient R2 had values of over 0.50, showing that these correlations are significant and adequately reproduce the connection between hardness and chemical composition;
- For values of the hardness parameter between 197 and 255 HB, the variation limits for the independent parameters result, namely in C = 2.97–3.15%, Mn = 0.53–0.65%, Si = 1.3–1.8%, and P = 0.85–1.02%. Sulfur varied between 0.08 and 0.09%, and the residual elements did not exceed 0.2%.

From the analysis of microstructures performed on the two different types of brake shoes, the following conclusions were obtained:

- From the micro-structural analysis of the two types of clogs, S1 and S2, a distribution of lamellar and very little semi-arched lamellar graphite (type Gl5—maximum 80% and Gl6—minimum 20%) resulted in the form of isolated separations of type Gr1 at no more than 12.5%;
- The ferrite present in Figure 32b and Figure 33b did not exceed 5%, which was present in the form of islands;
- The phosphorous eutectic was present on all samples with a lacy appearance and interdendritic separation, the content of which varied between 12.5 and 15.5% for the two microstructures;
- The verification of the relations obtained in Matlab with industrial data led to differences of −2.62% and +3.12%, respectively, which can be considered acceptable in the present case;
- In the case of the new charges analyzed, the micro-structural analysis was similar; for this batch, it was possible to analyze using high-performance equipment and higher magnification orders.

## Figures and Tables

**Table 1:** The chemical composition of phosphorous cast iron brake shoes [2].

Chemical Composition, [%]			
Carbon Total (C_t_)	Si	P	Mn
2.90–3.30	1.20–2.00	0.80–1.10	(1.72%S + 0.30%) − 1%

**Table 2:** The average chemical composition of the experimental batches.

Conditions	Values	Chemical Composition, [%]					C_equivalent,_ [%]	Si/C
		C	Si	Mn	P	S		
Experimental	maximum	3.30	1.95	0.70	1.08	0.12	4.24	0.59
	minimum	2.90	1.20	0.46	0.8	0.02	3.49	0.41
	medium	3.06	1.53	0.57	0.99	0.05	2.29	1.00

**Table 3:** The hardness of the brake shoe samples.

Values	According to Specifications No. 1/SFMR/SDT/2000 [HB]	Hardness Determined According to SR EN ISO 6506-1:2015, [HB]				
		HBs	HBd	HBss	HBsc	HBsd
maximum	255	255	255	248	230	255
minimum	197	197	198	197	198	200
medium	225	225	226	222	212	225

**Table 4:** Chemical composition of experimental charges.

Charge No.	Chemical Composition, [%]			
	C	Mn	Si	P
A1	3.03	1.36	0.60	0.99
A2	2.97	1.59	0.63	0.95
Recommended range	2.97–3.15	0.53–0.65	1.3–1.8	0.85–1.02

**Table 5:** Hardness samples of experimental charges.

Charge No.	HB				
	Brake Shoes Ends		Brake Shoes Section		
	Hs	Hd	Hss	Hsc	Hsd
A1	227	229	225	205	229
A2	224	228	226	207	228
Required range	197–255				

## Data Availability

The data presented in this study are available on request from the corresponding author.
